# Age-related changes in mice behavior and the contribution of lipocalin-2

**DOI:** 10.3389/fnagi.2023.1179302

**Published:** 2023-04-24

**Authors:** Ana Catarina Ferreira, Nuno Sousa, João Carlos Sousa, Fernanda Marques

**Affiliations:** ^1^Life and Health Sciences Research Institute (ICVS), School of Medicine, University of Minho, Campus Gualtar, Braga, Portugal; ^2^ICVS/3B’s—PT Government Associate Laboratory, Braga/Guimarães, Portugal

**Keywords:** aging, behavior, lipocalin-2, hippocampus, cell survival

## Abstract

Aging causes considerable changes in the nervous system, inducing progressive and long-lasting loss of physiological integrity and synaptic plasticity, leading to impaired brain functioning. These age-related changes quite often culminate in behavioral dysfunctions, such as impaired cognition, which can ultimately result in various forms of neurodegenerative disorders. Still, little is known regarding the effects of aging on behavior. Moreover, the identification of factors involved in regenerative plasticity, in both the young and aged brain, is scarce but crucial from a regenerative point of view and for our understanding on the mechanisms that control the process of normal aging. Recently, we have identified the iron-trafficking protein lipocalin-2 (LCN2) as novel regulator of animal behavior and neuronal plasticity in the young adult brain. On the other hand, others have proposed LCN2 as a biological marker for disease progression in neurodegenerative disorders such as Alzheimer’s disease and multiple sclerosis. Still, and even though LCN2 is well accepted as a regulator of neural processes in the healthy and diseased brain, its contribution in the process of normal aging is not known. Here, we performed a broad analysis on the effects of aging in mice behavior, from young adulthood to middle and late ages (2-, 12-, and 18-months of age), and in the absence of LCN2. Significant behavioral differences between aging groups were observed in all the dimensions analyzed and, in mice deficient in LCN2, aging mainly reduced anxiety, while sustained depressive-like behavior observed at younger ages. These behavioral changes imposed by age were further accompanied by a significant decrease in cell survival and neuronal differentiation at the hippocampus. Our results provide insights into the role of LCN2 in the neurobiological processes underlying brain function and behavior attributed to age-related changes.

## Introduction

Aging is a complex process that causes significant structural and physiological changes in the brain, often culminating in behavioral impairments and increased occurrence of neuropsychiatric and neurodegenerative disorders (Perna et al., 2016; Shoji et al., 2016). Several studies have, in fact, explored the impact of aging on brain physiology and behavior (Shoji et al., 2016; Botton et al., 2017), demonstrating age-related impairments on cognitive functions (Benice et al., 2006), exploratory and locomotor activities (Fahlstrom et al., 2011), sensorimotor behavior (Lau et al., 2008), and depressive-like states (Malatynska et al., 2012). Still, there are only few reports (Shoji et al., 2016) describing age-related changes in behavior, from young adulthood to middle and late ages.

Functional changes in the brain imposed by aging include the gradual loss of neural regenerative capacity. Specifically, in the hippocampus, morphological and functional changes associated with aging includes neuronal loss (Mattson and Magnus, 2006), decreased synaptic density (Bach et al., 1999; Barnes, 2003) and synaptogenesis (Geinisman et al., 1992), increased oxidative (Forster et al., 1996; Hu et al., 2006), and metabolic stress (Yin et al., 2016), and diminished neurogenesis (Kuhn et al., 1996; Kempermann et al., 2002). These changes strongly correlate with age-associated cognitive decline and memory impairments (Rapp and Heindel, 1994). Particularly, reduced adult hippocampal neurogenesis during aging was shown to be associated with decreased cognitive and learning performances (Lazarov et al., 2010; Mu and Gage, 2011; Merkley et al., 2014), which could be restored by increasing neurogenesis with exercise (van Praag et al., 2005; Merkley et al., 2014; Wu et al., 2015). In this sense, manipulations aimed at increasing neurogenesis constitute a promising approach for alleviating disease- or age-related impairments in brain function and behavior (Drew and Hen, 2007; Bolognin et al., 2014). Still, the nature of the causal factors responsible for neurodegeneration during aging are poorly understood. The identification of regulators that can control both hippocampal plasticity and function is crucial from a regenerative point of view, and for our understanding on the neurobiological mechanisms that underlie the aging process.

In recent years, the secreted protein lipocalin-2 (LCN2) was identified as a regulator of brain physiological process, by controlling neuronal structural and remodeling, synaptic activity and behavior at young adulthood (Mucha et al., 2011; Ferreira et al., 2013), with additional described roles in neural stem cells physiology and proliferation (Ferreira et al., 2018). On the other hand, LCN2 has also been described with important roles in the diseased brain, particularly in mild cognitive impairment (Choi et al., 2011), Alzheimer’s disease (Naude et al., 2012), multiple sclerosis (Marques et al., 2012) and, more recently, in Parkinson’s disease (Kim et al., 2016). Whether detrimental or protective (Ferreira et al., 2015), the described roles of LCN2 in brain homeostasis, along with its presence in brain regions affected by degeneration, has even proposed the usage of LCN2 as a biological marker for disease progression (Ferreira et al., 2015). Still, the contribution of LCN2 for the process of normal aging is currently unknown.

In this study, we performed mice behavioral analysis during aging, from young adulthood to middle and late ages (2-, 12-, and 18-months of age), and further analyzed the impact of LCN2 absence in the course of the behavioral effects related to aging. Moreover, we estimated cell survival and neuronal differentiation in the hippocampus as a measure of neural plasticity in the aged brain.

## Materials and methods

### Ethics statement

All animal procedures were conducted in accordance with the guidelines for the care and handling of laboratory animals in the Directive 2010/63/EU of the European Parliament and the Council and were approved by the Portuguese national authority for animal experimentation, Direção Geral de Alimentação e Veterinária (ID: DGAV9457). Animals were housed and maintained in a controlled environment at 22–24°C and 55% humidity, on 12 h light/dark cycles and fed with regular rodent’s chow and tap water *ad libitum*.

### Animal model and experimental groups

Experiments were conducted in male mice lacking LCN2 (LCN2-null), and the respective wild-type (Wt) littermate controls, in a C57BL/6 J mice background, obtained from heterozygous crossings. Mice were divided into three groups, accordingly to their age: 2-, 12- and 18-months. Both LCN2-null mice and age-matched Wt littermates were used for behavioral and cellular analysis. As a measure of general welfare of animals, body weight was monitored throughout the aging process.

### Behavior

**Figure 1:**
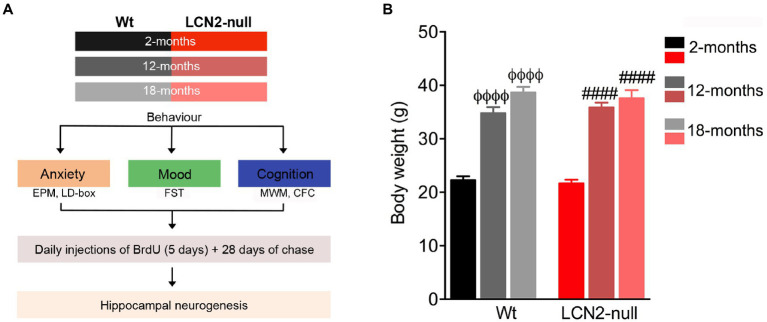
Body weight increases in aged animals, irrespectively of the genotype. **(A)** Schematic diagram of the experimental approach and the group of animals used at the respective ages. **(B)** Body weight measurements at selected ages revealed an age-related effect, with aged animals presenting increased body weight, when compared to genotype-matched 2-months old animals (*n* = 10–12 mice per group). Data are presented as mean ± SEM, analyzed by two-way analysis of variance (ANOVA) with Bonferroni’s multiple comparison test. ^Φ^Denotes differences between young and aged Wt; ^#^between young and aged lipocalin-2 (LCN2)-null mice. ^ΦΦΦΦ, ####^*p* ≤ 0.0001. EPM, elevated plus maze; CFC, contextual fear conditioning; FST, forced-swim test; MWM, Morris water maze.

Animals from two different cohorts, and at each selected age, were assessed for their behavior that included general evaluation of anxiety, mood and cognition (Figure 1A).

### Elevated plus maze

Anxious behavior was analyzed through the elevated plus maze (EPM) test. The behavioral apparatus (ENV-560; Med Associates Inc., St. Albans, VT, USA) consisted of two opposite open arms (50.8 cm Å ~ 10.2 cm) and two closed arms (50.8–10.2 cm Å ~ 40.6 cm), elevated 72.4 cm above the floor and dimly illuminated. Mice were individually placed in the center of the maze and allowed to freely explore it for 5 min. The percentage of time spent in the open arms, monitored through an infrared photobeam system (MedPCIV, Med Associates Inc.), was used as an index of anxiety-like behavior, and the number of total entries in the arms of the maze as an indicator of locomotor activity.

### Light/dark box test

Anxiety behavior was also assessed in the light/dark box test, consisting of an open field arena divided in equal parts. One part was open and brightly illuminated, with the other consisting of a black Plexiglas insert with an entrance at the center of the arena. Each animal was placed at the center of the arena facing the lateral wall and allowed to explore it for 10 min. An infrared automatic system (Med Associates Inc.) allowed monitoring the time spent in each compartment of the arena, and anxiety was calculated by the ratio of time spent in the dark versus the light compartment.

### Forced-swim test

Learned-helplessness was assessed through the forced-swim test (FST), as a measure of depressive-like behavior. The test was conducted by placing each animal individually in transparent cylinders filled with tap water (25°C; depth 30 cm) for a 5 min period. The trials were videotaped and manually scored using the EthoLog V 2.2 software (Ottoni, 2000). Learned-helplessness behavior was defined as an increase in immobility time and a decrease in the latency of time to immobility (in sec).

### Morris water maze

Cognitive function, by means of spatial reference memory, was evaluated using the Morris water maze (MWM) paradigm. The water maze consisted of a white circular pool (170 cm in diameter, 50 cm in height) filled with tap water (23°C; 25 cm of depth) and placed in a poorly lit room with extrinsic clues. The water tank was divided into four imaginary quadrants and a transparent escape platform (14 cm in diameter; 30 cm high), invisible to the animals, was placed in the center of one of the quadrants. Mice were randomly placed in the water facing the wall in each of the quadrants, and allowed to search for the hidden platform maintained in the same position during the 4 days of the acquisition. The trial was considered as concluded when the platform was reached within the limit time of 120 s. If failing to reach the platform within this time-period, animals were guided to the platform and allowed to stay in it for 30 s and an escape latency time of 120 s was registered. During the 4 days of the acquisition phase, each animal was given four trials per day. Trials were video-captured by a tracking system (Viewpoint, Champagne-au-Mont-d’Or, France) and the time required to reach the platform (latency of time) was recorded for the consecutive trials/days.

### Contextual fear conditioning

Contextual fear conditioning (CFC) was conducted for 2 days, as previously described (Ferreira et al., 2018), to assess fear memory. Briefly, on day 1, mice were placed in the conditioning chamber and received three pairings of light and a terminating shock (1 s, 0.5 mA), spared from each other with an interval of 20 s. On the following day, to test for conditioned fear to the training context, animals were placed in the same chamber for 3 min as before, but with no presentation of the conditioned stimulus, and the entire session was scored for freezing. Two hours after, animals were presented to a novel context, with no grid, black plastic inserts covering the floor and the walls of the chamber and scent with vanilla extract. Each mouse was placed into the novel context for 3 min and freezing was scored for the entire session. Freezing behavior was manually scored by a blind observer using the EthoLog V2.2 software and defined as the complete absence of motion for a minimum of 1 s. Parameters analyzed included the total percentage of time freezing in the context (A) and (B), and the index of discrimination between contexts as the ratio of percentage of time freezing (contexts A-B)/percentage of time freezing (contexts A + B).

### BrdU injections

In the end of the behavioral assessments, and prior to sacrifice (Figure 1A), animals at the described ages were intraperitoneally (i.p.) injected with BrdU (50 mg/kg; Sigma Aldrich, St. Louis, MO, USA) twice a day for 5 consecutive days, followed by a chase period of 28 days. This allowed the analysis of the effect of aging on the progeny of stem cells and progenitors survival.

### Tissue preparation and immunohistochemistry

For tissue processing, brains from anesthetized mice [i.p. mixture of ketamine hydrochloride (150 mg/kg) plus medetomidine (0.3 mg/kg)] were perfused transcardially with 0.9% cold saline, coronally sectioned in a cryostat (20 μm), and further processed for immunohistochemistry. For BrdU immunostaining, antigen retrieval by heat with citrate buffer (10 mM; Sigma) was performed, followed by DNA denaturation with HCl (Sigma) for 30 min. An additional blocking step with a solution of PBS 0.3% Triton X-100 (PBS-T; Sigma) and 10% normal fetal bovine serum (FBS; Invitrogen, Carlsbad, CA, USA) for 30 min at room temperature (RT) was also performed. Primary antibodies incubation, diluted in blocking solution, occurred overnight at RT and included rat anti-BrdU (1:100; Abcam, Cambridge, UK) and mouse anti-NeuN (1,200; Millipore, Billerica, MA, USA). The respective fluorescent secondary antibodies anti-rat and anti-mouse combined to Alexa 594 or to Alexa 488 (Invitrogen) were used to detect the respective primary antibodies at a dilution of 1:500 (in PBS-T) for 2 h at RT. To stain the nucleus, sections were incubated with 4′,6-diamidino-2-phenylindole (DAPI, 1:1000; Sigma), after which slides were mounted with Immu-Mount (ThermoFisher Scientific, Waltham, MA, United States). Fluorescence images of the dentate gyrus (DG) of the hippocampus were acquired using the Olympus Fluoview FV1000 confocal microscope (Olympus, Hamburg, Germany) and the number of double-positive cells further calculated using Olympus Fluoview FV1000 software (Olympus), and normalized for the respective area (mm^2^).

### Statistical analysis

All experiments were performed and analyzed by the same experimenter, blind to the animals’ genotype or group under assessment. Variables followed a Gaussian distribution as revealed by the D’Agostino & Pearson normality test. Data are reported as mean ± standard error (S.E.M.). The number of biological replicates (n) is specified in the legend of each figure. Statistically significant differences between groups were determined using two-way ANOVA, followed by Bonferroni’s multiple comparison test. Values were considered statistically significant for *p* ≤ 0.05 (*, # or Φ), *p* ≤ 0.01 (**, ## or ΦΦ), *p* ≤ 0.001(***, ### or ΦΦΦ) and *p* ≤ 0.0001(****, #### or ΦΦΦΦ).

## Results

### Depressive-like behaviors, but not anxiety, persists in aged LCN2-null mice

In order to assess the effects of aging in animal behavior, and in the absence of LCN2, Wt and LCN2-null mice were aged until 12- and 18-months of age, and evaluated for anxiety, depressive-like and cognitive behavioral dimensions (Figure 1A). Firstly, we examined age-related changes on the body weight of Wt and LCN2-null mice, and compared to young 2-months old mice. We observed a significant effect of age on body weight (*F*_2,60_ = 142.0, *p* < 0.0001), independently of the genotype (genotype*age: *F*_2,60_ = 0.53, *p* = 0.59; Figure 1B). Both aged Wt and LCN2-null mice were significantly heavier than younger genotype-matched animals (12- and 18-months >2-months, *p* < 0.0001; Figure 1B).

**Figure 2:**
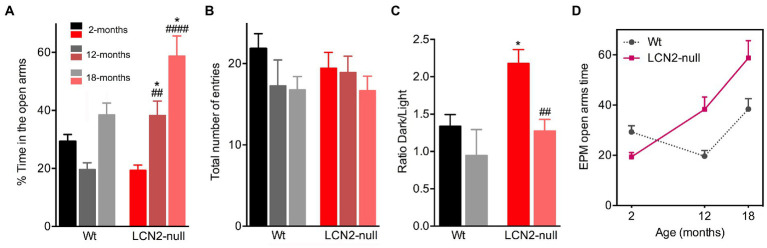
Age-related alterations of anxiety-like behaviors in Wt and lipocalin-2 (LCN2)-null mice. **(A)** Assessment of anxiety-like behavior in the elevated plus maze (EPM) showed an increase in the percentage of time spent in the open arms across age, specifically in LCN2-null mice (*n* = 10–12 mice per group). **(B)** General locomotor activity assessed by the total number of entries in the maze revealed no major aging and genotype effects. **(C)** In the light/dark box test, anxiety-like behavior of LCN2-null mice at 2-months of old is reduced at 18-months of age. **(D)** Representative progression of EPM performance by the animals during the course of normal aging, evidencing the reduction in anxiety behavior (as an increased time in the EPM open arms) observed in LCN2-null mice. At 12-months of age, Wt mice become more anxious, a phenotype that was lost at 18-months. Data are presented as mean ± SEM, analyzed by two-way ANOVA with Bonferroni’s multiple comparison test. ^#^Denotes differences between young and aged LCN2-null mice; *between Wt and LCN2-null mice at each matched age. ^*^p ≤ 0.05, ^##^*p* ≤ 0.01, ^####^*p* ≤ 0.0001.

To assess anxiety-like behaviors, we tested the animals of both genotypes and at the different ages in the EPM test. Analysis of the effect of age in the time spent in the open arms revealed that it significantly influenced animal’s performance in the maze (age effect: *F*_2,52_ = 16.05, *p* < 0.0001). Specifically, Wt 12-months old animals spent less time in the open arms of the maze (12- < 2-months, *p* = 0.59), which suggested the appearance of an anxious-like behavior with aging. However, at the oldest age of 18-months, Wt animals exhibited a surprisingly increased percentage of time spent in the open arms (18- > 12-months, *p* = 0.07; Figure 2A). In addition, aging also induced a reduction in the anxiety state presented by LCN2-null mice at 2-months of age. We have previously described that young LCN2-null mice present an increased anxiety-like behavior in the EPM test (Ferreira et al., 2013), which we confirmed here (Wt: 29%, LCN2-null: 18%; Figure 2A). Analysis of aged LCN2-null mice in the EPM showed that animals significantly increased the time spent in the open arms of the maze, when compared to younger mice (12- > 2-months, *p* = 0.006; 18- > 2-months, *p* < 0.0001), and to age-matched Wt animals (Wt versus LCN2-null: 12-months, *p* = 0.05; 18-months, *p* = 0.01; Figure 2A).

In addition, the evaluation of the total number of entries in the EPM maze, as a measure of general locomotor activity, revealed no aging effect in this parameter (F_1,29_ = 1.16, *p* = 0.29), neither between ages or genotypes (Figure 2B). To confirm the observations obtained in the time spent in the open arms of the EPM maze, we also tested the animals, but only at 18-months of age in the light/dark box test, another commonly used test to assess anxiety-like behavior. Indeed, a similar phenotype was observed when testing 18-months old Wt and LCN2-null mice in the light/dark box test. In this test, a decreased ratio time dark/light was observed in older LCN2-null animals, when compared to younger mice (18- < 2-months, *p* = 0.002; Figure 2C), confirming the reduction in anxiety by aging in LCN2-null mice. For aged Wt mice, this was not evident (*p* = 0.67; Figure 2C). Overall, these results suggest that, along with the process of aging, a reduction in anxiety behavior occurs in the absence of LCN2 (Figure 2D).

**Figure 3:**
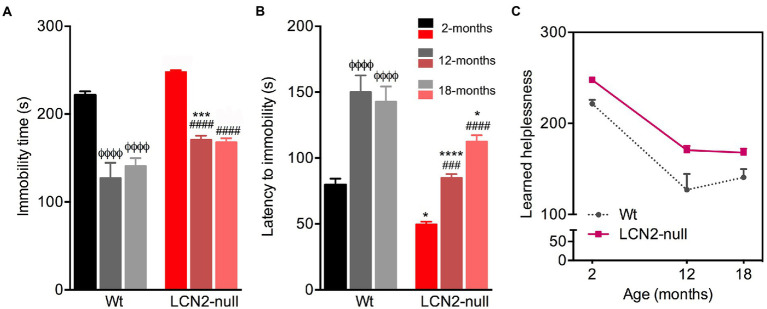
Wt and lipocalin-2 (LCN2)-null mice depressive-like behavior in the course of aging. **(A)** Depressive-like behavior evaluated as learned helplessness in the forced-swim test (FST) revealed decreased immobility time in older Wt and LCN2-null mice, with null aged mice continuing to present a depressive-like behavior (*n* = 10–12 mice per group). **(B)** Mood alterations in older mice were also observed by the latency of time to immobility. **(C)** Depressive-like behavior in the progress of aging remains in LCN2-null mice, compared to controls, while older Wt animals decreased their immobility time. Data are presented as mean ± SEM, analyzed by two-way ANOVA with Bonferroni’s multiple comparison test. ^Φ^Denotes differences between young and aged Wt mice; ^#^between differences between young and aged LCN2-null mice; *between Wt and LCN2-null mice at each matched age. ^*^p ≤ 0.05, ^##^*p* ≤ 0.01, ^***^p ≤ 0.001, ^ΦΦΦΦ, ####, ****^p ≤ 0.0001.

Following, we assessed learned helplessness as an index of depressive-like behavior, by the immobility time and the latency to immobility in the FST. Aging had an overall significant effect on the immobility time (*F*_2,41_ = 71.96, *p* < 0.0001) and the latency to immobility (*F*_2,37_ = 33.56, *p* < 0.0001; Figures 3A,B) presented by the animals. Older Wt mice significantly decreased their immobility time in the FST, when compared to 2-months old mice (2- > 12-, 18-months, *p* < 0.0001; Figure 3A). In line with this, aged Wt animals also increased their latency of time to immobility (2- < 12-, 18-months, *p* < 0.0001), suggesting that aging does not promote a depressive-like behavior (Figure 3B). Notably, aged LCN2-null mice also decreased the time spent immobile, when comparing to 2-months old null mice (2- > 12-, 18-months, *p* < 0.0001; Figure 3A), and increased their latency to immobility (2- > 12-months, *p* = 0.0005; 2- > 18-months, *p* < 0.0001; Figure 3B). Still, animals presented a depressive-like phenotype when compared to Wt age-matched animals (immobility time—Wt versus LCN2-null: 12-months, *p* = 0.0005; 18-months, *p* = 0.09; latency—Wt versus LCN2-null: 12-months, *p* < 0.0001; 18-months, *p* = 0.05).

Together, this suggests that learned helplessness in the FST revealed an age-related decrease in depressive-like behavior in both group of animals, but still LCN2-null mice sustained a depressive-like behavior across the aging process (Figure 3C).

### Spatial learning and contextual discrimination are affected through the course of aging

**Figure 4:**
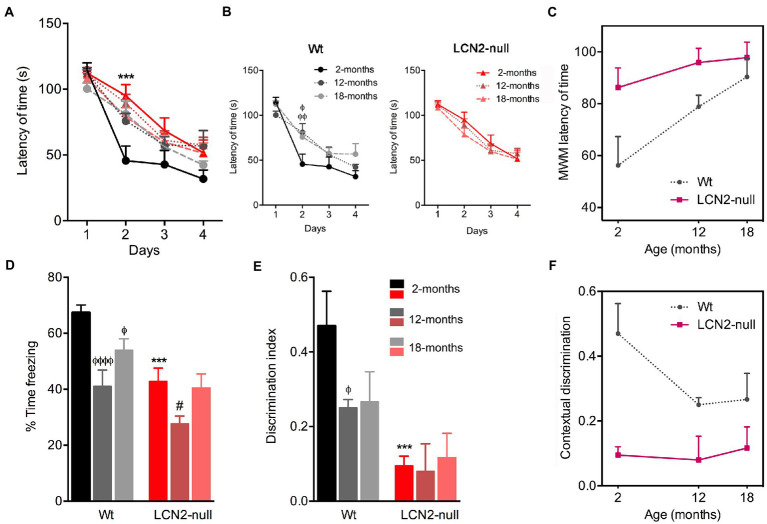
Spatial learning and memory retrieval and consolidation decreases during normal aging. **(A)** Analysis of spatial learning and memory in the Morris water maze (MWM) test revealed that all groups learned to find the position of the hidden platform across the 4 days of acquisition. **(B)** Older Wt mice required more time to find the platform, while lipocalin-2 (LCN2)-null animals presented similar impaired learning curves throughout aging (*n* = 10–12 mice per group). **(C)** Cognitive deficits in the course of normal aging, measured by the reduced latency of time in the MWM. **(D)** Freezing behavior upon re-exposure to conditioning context revealed a major effect of aging in Wt animals, as freezing behavior decreased in older animals. **(E)** Impaired memory retrieval and consolidation was confirmed by the decreased ratio of discrimination index in aged Wt mice, which was sustained in LCN2-null mice throughout aging (n = 10–12 mice per group). **(F)** Impaired contextual discrimination along aging in Wt and LCN2-null mice. Data are presented as mean ± SEM, analyzed by two-way ANOVA with Bonferroni’s multiple comparison test. ^Φ^Denotes differences between young and aged Wt mice; ^#^between differences between young and aged LCN2-null mice; ^*^between Wt and LCN2-null mice at each matched age. ^Φ, #^*p* ≤ 0.05, ^ΦΦΦΦ^*p* ≤ 0.0001, ^***^p ≤ 0.001.

To examine age-related changes in spatial learning and memory, we tested the animals, at the different ages, in the MWM paradigm. In this task, all animals, regardless of the genotype, learned to find the position of the hidden platform, as they improved the time required to find it along the 4 days of the test (Figure 4A). The only difference obtained concerned the behavioral performance of 2-months old LCN2-null mice on the day 2 of testing (*p* = 0.0002), in accordance to what we have previously reported (Ferreira et al., 2013). Moreover, the specific analysis of the learning process of Wt animals revealed that it was affected by aging (*F*_5,53_ = 3.06, *p* = 0.02). We observed a higher latency of time required by 12- and 18-months old Wt mice to reach the hidden platform, more evident on the second day of testing (12- > 2-months, *p* = 0.02; 18- > 2-months, *p* = 0.005; Figure 4B). On the other hand, the learning profile of LCN2-null mice remained similar across ages (*F*_2,33_ = 0.69, *p* = 0.51; Figure 4B): the observed compromised spatial learning of LCN2-null mice at 2-months old persisted throughout the aging process (Figure 4B). Although aging induced memory and learning deficits in Wt mice, LCN2-null animals required always increased time to find the platform in the MWM task (Figure 4C). Altogether, the increased latency of time in the MWM indicates that spatial learning performances decreased during aging in Wt conditions, remaining poorer in LCN2-null mice (as depicted in Figure 4C).

Additionally, to the MWM test, we have also used the CFC to assess contextual discrimination, as a measure of fear memory. After training the animals to a cued conditioning task, animals were re-exposed to the conditioning context and freezing behavior was scored as a measure of memory contextual retrieval. Analysis of the effect of age in the freezing time revealed that it significantly influenced animal’s performance (age effect: *F*_2,34_ = 20.83, *p* < 0.0001). Specifically, we observed that older Wt animals significantly decreased their freezing behavior, in comparison to younger animals (12- < 2-months, *p* < 0.0001; 18- < 2-months, *p* = 0.02; Figure 4D). On the other hand, freezing behavior of LCN2-null mice remained similar throughout ages. The decreased freezing behavior observed already at 2-months old (Wt versus LCN2-null, *p* = 0.0005) persisted in aged null animals [more pronounced in 12-months animals (12- < 2-months, *p* = 0.02; Figure 4D)]. In addition, analysis of contextual discrimination index, after presentation of a novel context, revealed that aging affected discrimination indexes. Similarly, for contextual retrieval, aging significantly decreased the index of contextual discrimination in Wt animals (12- < 2-months, *p* = 0.05; Figures 4E,F), while LCN2-null mice presented the same ratios of contextual discrimination across ages, similar to younger animals (Wt versus LCN2-null: 2-months, *p* = 0.001; Figure 4E). Aging significantly affected the ability of animals to discriminative different contexts presentation, thus influencing fear memory (Figure 4F).

### The generation of newborn neurons in the hippocampus decreases with aging

**Figure 5:**
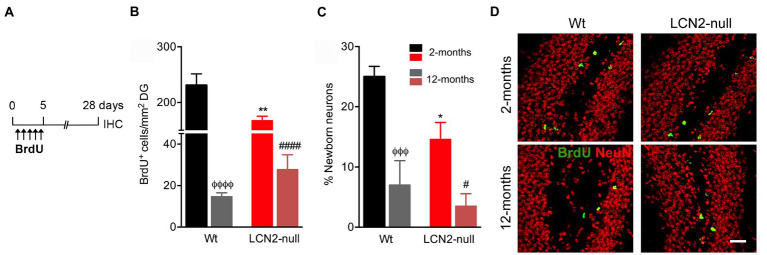
Aging reduces hippocampal neurogenesis in both Wt and lipocalin-2 (LCN2)-null animals. **(A)** Schematic diagram of the BrdU protocol used to label cell survival and neuronal differentiation. **(B)** Quantification of total number of BrdU^+^ cells in the DG of Wt and LCN2-null mice disclosed a significant effect of aging in cell survival (*n* = 4–5 mice per group). **(C)** Percentage of newly born neurons are significantly affected by aging, independently of animal’s genotype. **(D)** Representative images of BrdU and NeuN staining in the DG of Wt and LCN2-null mice. Data are presented as mean ± SEM, analyzed by two-way ANOVA with Bonferroni’s multiple comparison test. ^Φ^Denotes differences between young and aged Wt mice; #between differences between young and aged LCN2-null mice; ^*^between Wt and LCN2-null mice at each matched age. ^#,*^p ≤ 0.05, ^**^p ≤ 0.01, ^ΦΦΦ^*p* ≤ 0.001; ^ΦΦΦΦ, ####^*p* ≤ 0.0001. IHC, immunohistochemistry.

In order to disclose the contribution of hippocampal plasticity to the described age-related alterations in behavior, in both Wt and LCN2-null mice, we next analyzed the generation of adult newborn neurons in the DG of the hippocampus. With this purpose, animals at the described ages were daily injected with BrdU for 5 days, followed by a chase period of 28 days (Figure 5A). Density of BrdU^+^ cells in the DG, as a mean of cell survival, revealed a significant effect of aging (*F*_1,13_ = 246.3, *p* < 0.0001). Animals at 12-months of age presented a significant decrease in the number of labeled survival cells (Figure 5B). Both Wt and LCN2-null mice, in comparison to younger animals, presented a significant decreased in the total number of BrdU^+^ cells in the DG (Wt: 12- < 2-months, *p* < 0.0001; LCN2-null mice: 12- < 2-months, *p* < 0.0001; Figure 5B). Despite the observed significant decreased cell survival in LCN2-null animals at 2-months (Wt versus LCN2-null, *p* = 0.002), it was evident that this cell population was similarly affected during the course of the aging process, as in the Wt animals.

Additionally, analysis of the percentage of newborn neurons generated in the DG, within the 28-day period of labeling, showed that aging significantly impaired the formation of new neurons (F_1,14_ = 28.50, *p* < 0.0001). Specifically, aged Wt mice presented a significant reduction in the percentage of newborn neurons (12- < 2-months, *p* = 0.0007; Figure 5C), similarly observed in LCN2-null mice at 12-months of age (12- < 2-months, *p* = 0.02; Figure 5C). Again, despite the observed impairments at 2-months in LCN2-null mice (Wt versus LCN2-null, *p* = 0.02), cell differentiation was significantly affected by aging.

## Discussion

**Figure 6:**
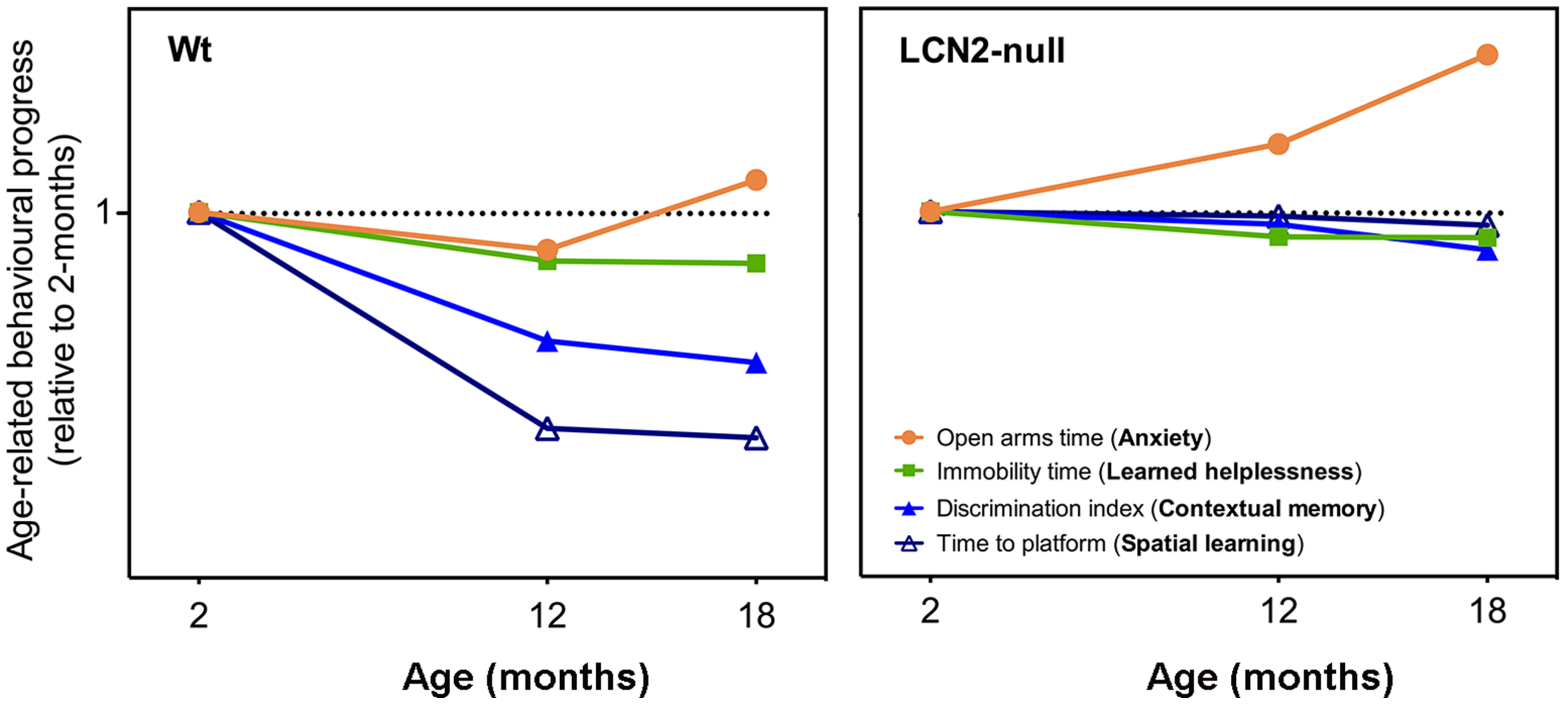
Schematic representation on the progression of the behavioral dimensions assessed throughout aging, in both Wt and lipocalin-2 (LCN2)-null mice. Comparison of behavioral performances of aged animals with younger ages revealed that aging, in Wt mice, slightly decreased their anxiety state at 18-months (as observed by the increased open arms time), but did not promote depressive-like behaviors (immobility time is decreased). Cognitive domains, both spatial learning and contextual discrimination, was significantly impaired with aging. On the other hand, aged LCN2-null mice sustained their impaired behavior observed already at 2-months of age, specifically in depressive-like behavior and cognitive domains, with the exception of anxiety that was significantly decreased (represented as the increased in the time spent in the open arms).

In the present study, we evaluated the impact of aging in several dimensions of animal behavior, including mood, anxiety and cognition, and upon the absence of LCN2. Moreover, we evaluated brain plasticity in the form of hippocampal neurogenesis as one of the neurobiological mechanisms playing a more determining role in the observed behavioral outcomes across aging. Our results indicate that aged Wt animals presented progressive cognitive deficits, in both contextual discrimination and spatial learning, but not of anxiety and learned helplessness, when comparing to behavioral performances at younger ages (Figure 6). Similarly, the effects of LCN2 absence in learned helplessness and cognition, already observed at 2-months of age (Ferreira et al., 2013), were sustained throughout aging (Figure 6). The exception concerned the anxiety domain, since the time spent in the open arms by LCN2-null mice increased with aging (Figure 6). Moreover, age also significantly impacted on hippocampal neurogenesis, in both Wt and LCN2-null mice, as seen by the prominent decrease in cell survival and in the generation of new neurons.

Evaluation of anxiety-like behavior along the process of aging revealed that 12-months old Wt mice present an anxious-like behavior, when compared to younger mice. However, at later ages, a reduced anxiety was observed (Figure 6). This reduction was independent of LCN2, since aged LCN2-null mice, at both EPM and light/dark box tests, decreased the time spent in the open arms and in the light compartment, respectively. Although not so evident in aged Wt mice (Figure 6), our observations are similar to what others have also reported when analyzing age-related changes in C57BL/6 J mice behavior (Shoji et al., 2016; Botton et al., 2017). Particularly for the EPM, some authors speculate that the decrease in the time spent in the open arms of the maze, as we demonstrated here for our LCN2-null mice aging cohort, may reflect an increased panic-like escape response to the novel environment (Holmes et al., 2000; Hattori et al., 2012). Moreover, anxiety-like behavior assessments in aged mice are described to be test-dependent: a same cohort of aged animals, that were less anxious in the EPM, showed aversive behavior to the center area of the open field test (Botton et al., 2017). These differences have been attributed to the sensitivity of anxiety responses inherent to each behavioral apparatus, which also varies with age (Botton et al., 2017). Nevertheless, here we observed the same phenotype on both EPM and light/dark box test (at least for the 18-months timepoint) in LCN2-null animals.

Regarding depressive-like behavioral domain, while some hypothesis in the literature suggest that aging is associated with an increased risk of depression (Hattori et al., 2012), some inconsistences are also reported on the effects of aging in this type of behavior (Godbout et al., 2008; Malatynska et al., 2012; Shoji et al., 2016). The described contradictions have been mainly attributed to the different ages used, animal species and strains, or even to behavioral procedures. While some described a lack of affective deficits in 18-months old C57BL/6 mice in the FST (Malatynska et al., 2012), others reported that immobility time in the FST decreases from young adulthood to middle age [8–12-months old; (Shoji et al., 2016)]. In fact, our results are in agreement, as we observed decreased immobility time at 12- and 18-months of age, when compared to the younger ages (Figure 6), in both Wt and LCN2-null mice. This decrease might be explained by the fact that the same animals also decreased their anxiety across ages (although only evident in Wt mice at 18-months). In addition, several factors are able to influence FST performance (Bogdanova et al., 2013) as for instance the levels of corticosterone in the day of the test (Martínez-Mota et al., 2011). In addition, it was previously suggested that FST is a suitable model to evaluate depressive-like behaviors in prepubertal rats and that the changes in corticosterone and gonadal hormones could mediate the sex and age differences in the behavioral responses in the FST (Martínez-Mota et al., 2011). It is well known that increased anxiety is an important feature of depressive states, and the observed decreased anxiety, at least for the LCN2-null mice, may account to the reduced behavioral despair observed in the FST by aged mice. Still, and even though the immobility time in LCN2-null mice decreased with age, learned helplessness persisted in aged mice (Figure 6) when compared to aged-matched Wt mice, which may suggest a putative involvement of LCN2 in late depression. The prevalence of depression is elevated in older people (Glaesmer et al., 2011) and, taking into consideration our results, aged LCN2-null mice could constitute an effective animal model to explore putative novel therapeutic approaches in late-life depression. In fact, increased LCN2 plasma levels were observed in depressed older patients, proposing LCN2 as a marker in the pathophysiology of late-life depression (Naude et al., 2013).

One prominent feature associated with aging, and extensively explored and reported in aged animal models, concerns the progressive loss of cognitive functions, especially in certain types of learning and memory (Bach et al., 1999). In line with other reports (Magnusson et al., 2003; Bergado et al., 2011; Shoji et al., 2016), we here observed, in Wt mice, an aged-related spatial learning and memory defect in the MWM task (Figure 6). In contrast, LCN2-null mice performance was sustained throughout aging (Figure 6). The very mild behavioral performance presented by LCN2-null mice at 2-months of age (Ferreira et al., 2013) remained the same, regardless of the aging process. Of interest the impairment of the LCN2-null mice in the hippocampal spatial learning and memory is very mild and only verified at day 2 of the MWM training phase protocol. This data is also in accordance with Dekens et al., in which it was shown that, LCN2-null mice showed significantly slower MWM learning curves compared to WT mice, possibly indicating mild memory impairment during the training phase (Dekens et al., 2018). In addition, it was previously demonstrated, that LCN2-null mice performed similar to WT mice in the first and second probe trial and so, we cannot describe here an alteration in memory (Ferreira et al., 2013; Dekens et al., 2018). Moreover, when tested for contextual fear memory, a more DG-dependent task, age-related deficits in the ability to discriminate between contexts was observed in both genotypes. With age, Wt mice decreased their contextual discrimination, as observed by the impaired discrimination indexes, while LCN2-null mice, similarly to the MWM performance, presented a constant impaired contextual discrimination across aging (Figure 6). These findings are, in fact, relevant and should be contemplated when considering the usage of LCN2 levels as predictors of cognitive impairments. The description of increased LCN2 plasma levels during mild-cognitive impairment, and the consequent suggestion that it might be helpful in predicting the progression of this state to Alzheimer’s disease (Choi et al., 2011), should be considered with caution in light of the present results.

Associated with age-related poor cognitive performances are also the descriptions of functional changes in the hippocampus, the brain region mostly involved in learning and memory (Jarrard, 1995). Alterations include hippocampus structural atrophy (Small et al., 2002) and decreased volume (Mattson and Magnus, 2006), as well as reduced hippocampal neurogenesis (Kuhn et al., 1996) and synaptic plasticity (Barnes, 1994). In fact, deficits in hippocampal long-term potentiation (LTP) imposed by aging have been correlated with defects in spatial memory (Bach et al., 1999; Barnes, 2003). In this sense, it is interest to observe that LCN2-null mice, at 2-months of age, were described to present neuronal atrophy in the dorsal hippocampus and synaptic impairments in hippocampal LTP (Ferreira et al., 2013). Even though we do not know if this is the case, these differences may be accounting to the sustained impaired cognitive function described. We can only speculate that these impairments might persist until older ages and, therefore, contribute for the cognitive decline maintenance in these animals. Nevertheless, further electrophysiological assessments and morphological reconstructions would be required to confirm this. Other signs of age-associate decline in hippocampal plasticity include hippocampal neurogenesis, which diminishes with aging (Kuhn et al., 1996; Heine et al., 2004). Hippocampal neurogenesis largely contributes to cognition and memory and, in fact, age-related decreased hippocampal neurogenesis has been suggested as the basis for learning and memory decline (Bizon et al., 2004) and impaired contextual discrimination (Moyer and Brown, 2006) during aging. In accordance, our descriptions of impaired learning and cognition and fear memory in aging were further accompanied by a decreased cell survival and neuronal differentiation in the hippocampus of aged mice. Herein, we only performed the analysis of the newborn neurons in 2- and 12-month-old mice, which is certainly a limitation of the study, this is certainly a great contributor to the observed impaired phenotypes. However, in the future, the quantification of newborn neurons also in the 18-month-old mice, to correlate the information with the behavioral outcomes, will be relevant. Moreover, we have recently reported LCN2 to be an important regulator of hippocampal neurogenesis (Ferreira et al., 2018). In young LCN2-null mice, decreased neurogenesis contributes to impaired contextual discriminative behaviors (Ferreira et al., 2018). Still, and although the generation of newborn neurons similarly decreased in aged LCN2-null DG, as in the Wt, this did not translate into a worsen cognitive phenotype. Probably, in this case, is not just a matter of down-regulated neurogenesis but rather of impaired functionality of the new neurons added into the hippocampal circuitry. Moreover, oxidative stress imposed by the absence of LCN2, as we have previously described to impair neurogenesis and contextual discrimination at younger ages (Ferreira et al., 2018), may also account for the sustained impaired behavior in aging. Oxidative stress is also considered to underlie aging-related cognitive impairments and degeneration (Nicolle et al., 2001; Hu et al., 2006).

## Concluding remarks

With the average age of the world’s population rapidly rising (United Nations World Population Aging, 2013), the need for studies investigating aging-related cognitive impairments has become increasingly important. In the literature, the usage of animal models of aging has proven useful not only for understanding the aging process, but also for understanding the functioning of the hippocampus. In addition, the plasticity and regeneration capacity intrinsic to the hippocampus, in part explained by the neurogenesis process, opens novel perspectives on the neurobiology of aging. We believe that, with the present report, we have contributed to this field, by describing the behavioral performance of Wt mice during the process of aging, in addition to the elucidation on how a single protein involved in plasticity modulation contributes to such process.

## Data availability statement

The raw data supporting the conclusions of this article will be made available by the authors, without undue reservation.

## Ethics statement

The animal study was reviewed and approved by the Portuguese national authority for animal experimentation, Direção Geral de Alimentação e Veterinária (ID: DGAV9457) and are in accordance with the guidelines for the care and handling of laboratory animals in the Directive 2010/63/EU of the European Parliament and the Council.

## Author contributions

AF performed the experiments and wrote the first draft. JS performed the experiments. FM and NS revision and editing of the manuscript. All authors contributed to the article and approved the submitted version.

## Funding

This work has been funded by National funds, through the Foundation for Science and Technology (FCT)—project UIDB/50026/2020 and UIDP/50026/2020 and by National funds, through the Foundation for Science and Technology (FCT)—project UIDB/50026/2020 and UIDP/50026/2020 and by the project NORTE-01-0145-FEDER-000039, supported by Norte Portugal Regional Operational Program (NORTE 2020), under the PORTUGAL 2020 Partnership Agreement, through the European Regional Development Fund (ERDF).

## Conflict of interest

The authors declare that the research was conducted in the absence of any commercial or financial relationships that could be construed as a potential conflict of interest.

## Publisher’s note

All claims expressed in this article are solely those of the authors and do not necessarily represent those of their affiliated organizations, or those of the publisher, the editors and the reviewers. Any product that may be evaluated in this article, or claim that may be made by its manufacturer, is not guaranteed or endorsed by the publisher.

## References

[ref1] Bach M. E. Barad M. Son H. Zhuo M. Lu Y. F. Shih R.. (1999). Age-related defects in spatial memory are correlated with defects in the late phase of hippocampal long-term potentiation in vitro and are attenuated by drugs that enhance the cAMP signaling pathway. Proc. Natl. Acad. Sci. U. S. A. 96, 5280–5285. doi: 10.1073/pnas.96.9.5280, PMID: 10220457 PMC21855

[ref2] Barnes C. A. (1994). Normal aging: regionally specific changes in hippocampal synaptic transmission. Trends Neurosci. 17, 13–18. doi: 10.1016/0166-2236(94)90029-9, PMID: 7511843

[ref3] Barnes C. A. (2003). Long-term potentiation and the ageing brain. Philos. Trans. R. Soc. Lond. Ser. B Biol. Sci. 358, 765–772. doi: 10.1098/rstb.2002.1244, PMID: 12740124 PMC1693160

[ref4] Benice T. S. Rizk A. Kohama S. Pfankuch T. Raber J. (2006). Sex-differences in age-related cognitive decline in C57BL/6J mice associated with increased brain microtubule-associated protein 2 and synaptophysin immunoreactivity. Neuroscience 137, 413–423. doi: 10.1016/j.neuroscience.2005.08.029, PMID: 16330151

[ref5] Bergado J. A. Almaguer W. Rojas Y. Capdevila V. Frey J. U. (2011). Spatial and emotional memory in aged rats: a behavioral-statistical analysis. Neuroscience 172, 256–269. doi: 10.1016/j.neuroscience.2010.10.064, PMID: 21036203

[ref6] Bizon J. L. Lee H. J. Gallagher M. (2004). Neurogenesis in a rat model of age-related cognitive decline. Aging Cell 3, 227–234. doi: 10.1111/j.1474-9728.2004.00099.x, PMID: 15268756

[ref7] Bogdanova O. V. Kanekar S. D'Anci K. E. Renshaw P. F. (2013). Factors influencing behavior in the forced swim test. Physiol. Behav. 118, 227–239. doi: 10.1016/j.physbeh.2013.05.012, PMID: 23685235 PMC5609482

[ref8] Bolognin S. Buffelli M. Puolivali J. Iqbal K. (2014). Rescue of cognitive-aging by administration of a neurogenic and/or neurotrophic compound. Neurobiol. Aging 35, 2134–2146. doi: 10.1016/j.neurobiolaging.2014.02.017 24702821

[ref9] Botton P. H. Pochmann D. Rocha A. S. Nunes F. Almeida A. S. Marques D. M.. (2017). Aged mice receiving caffeine since adulthood show distinct patterns of anxiety-related behavior. Physiol. Behav. 170, 47–53. doi: 10.1016/j.physbeh.2016.11.030, PMID: 27890589

[ref10] Choi J. Lee H. W. Suk K. (2011). Increased plasma levels of lipocalin 2 in mild cognitive impairment. J. Neurol. Sci. 305, 28–33. doi: 10.1016/j.jns.2011.03.023, PMID: 21463871

[ref11] Dekens D. W. Naudé P. J. W. Keijser J. N. Boerema A. S. De Deyn P. P. Eisel U. L. M. (2018). Lipocalin 2 contributes to brain iron dysregulation but does not affect cognition, plaque load, and glial activation in the J20 Alzheimer mouse model. J. Neuroinflammation 15:330. doi: 10.1186/s12974-018-1372-5, PMID: 30501637 PMC6267886

[ref12] Drew M. R. Hen R. (2007). Adult hippocampal neurogenesis as target for the treatment of depression. CNS Neurol. Disord. Drug Targets 6, 205–218. doi: 10.2174/187152707780619353 17511617

[ref13] Fahlstrom A. Yu Q. Ulfhake B. (2011). Behavioral changes in aging female C57BL/6 mice. Neurobiol. Aging 32, 1868–1880. doi: 10.1016/j.neurobiolaging.2009.11.003 20005598

[ref14] Ferreira A. C. Da Mesquita S. Sousa J. C. Correia-Neves M. Sousa N. Palha J. A.. (2015). From the periphery to the brain: Lipocalin-2, a friend or foe? Prog. Neurobiol. 131, 120–136. doi: 10.1016/j.pneurobio.2015.06.005 26159707

[ref15] Ferreira A. C. Pinto V. Da Mesquita S. Novais A. Sousa J. C. Correia-Neves M.. (2013). Lipocalin-2 is involved in emotional behaviors and cognitive function. Front. Cell. Neurosci. 7:122. doi: 10.3389/fncel.2013.00122, PMID: 23908604 PMC3725407

[ref16] Ferreira A. C. Santos T. Sampaio-Marques B. Novais A. Mesquita S. D. Ludovico P.. (2018). Lipocalin-2 regulates adult neurogenesis and contextual discriminative behaviours. Mol. Psychiatry 23, 1031–1039. doi: 10.1038/mp.2017.95, PMID: 28485407

[ref17] Forster M. J. Dubey A. Dawson K. M. Stutts W. A. Lal H. Sohal R. S. (1996). Age-related losses of cognitive function and motor skills in mice are associated with oxidative protein damage in the brain. Proc. Natl. Acad. Sci. U. S. A. 93, 4765–4769. doi: 10.1073/pnas.93.10.4765, PMID: 8643477 PMC39353

[ref18] Geinisman Y. deToledo-Morrell L. Morrell F. Persina I. S. Rossi M. (1992). Structural synaptic plasticity associated with the induction of long-term potentiation is preserved in the dentate gyrus of aged rats. Hippocampus 2, 445–456. doi: 10.1002/hipo.450020412, PMID: 1308201

[ref19] Glaesmer H. Riedel-Heller S. Braehler E. Spangenberg L. Luppa M. (2011). Age- and gender-specific prevalence and risk factors for depressive symptoms in the elderly: a population-based study. Int. Psychogeriatr. 23, 1294–1300. doi: 10.1017/S1041610211000780, PMID: 21729425

[ref20] Godbout J. P. Moreau M. Lestage J. Chen J. Sparkman N. L. O'Connor J.. (2008). Aging exacerbates depressive-like behavior in mice in response to activation of the peripheral innate immune system. Neuropsychopharmacology 33, 2341–2351. doi: 10.1038/sj.npp.1301649, PMID: 18075491 PMC2907915

[ref21] Hattori S. Takao K. Tanda K. Toyama K. Shintani N. Baba A.. (2012). Comprehensive behavioral analysis of pituitary adenylate cyclase-activating polypeptide (PACAP) knockout mice. Front. Behav. Neurosci. 6:58. doi: 10.3389/fnbeh.2012.00058, PMID: 23060763 PMC3462416

[ref22] Heine V. M. Maslam S. Joels M. Lucassen P. J. (2004). Prominent decline of newborn cell proliferation, differentiation, and apoptosis in the aging dentate gyrus, in absence of an age-related hypothalamus-pituitary-adrenal axis activation. Neurobiol. Aging 25, 361–375. doi: 10.1016/S0197-4580(03)00090-3, PMID: 15123342

[ref23] Holmes A. Parmigiani S. Ferrari P. F. Palanza P. Rodgers R. J. (2000). Behavioral profile of wild mice in the elevated plus-maze test for anxiety. Physiol. Behav. 71, 509–516. doi: 10.1016/s0031-9384(00)00373-5, PMID: 11239669

[ref24] Hu D. Serrano F. Oury T. D. Klann E. (2006). Aging-dependent alterations in synaptic plasticity and memory in mice that overexpress extracellular superoxide dismutase. J. Neurosci. 26, 3933–3941. doi: 10.1523/JNEUROSCI.5566-05.2006, PMID: 16611809 PMC6673899

[ref25] Jarrard L. E. (1995). What does the hippocampus really do? Behav. Brain Res. 71, 1–10. doi: 10.1016/0166-4328(95)00034-8 8747170

[ref26] Kempermann G. Gast D. Gage F. H. (2002). Neuroplasticity in old age: sustained fivefold induction of hippocampal neurogenesis by long-term environmental enrichment. Ann. Neurol. 52, 135–143. doi: 10.1002/ana.10262, PMID: 12210782

[ref27] Kim B. W. Jeong K. H. Kim J. H. Jin M. Kim J. H. Lee M. G.. (2016). Pathogenic upregulation of glial Lipocalin-2 in the parkinsonian dopaminergic system. J. Neurosci. 36, 5608–5622. doi: 10.1523/JNEUROSCI.4261-15.2016, PMID: 27194339 PMC6601774

[ref28] Kuhn H. G. Dickinson-Anson H. Gage F. H. (1996). Neurogenesis in the dentate gyrus of the adult rat: age-related decrease of neuronal progenitor proliferation. J. Neurosci. 16, 2027–2033. doi: 10.1523/JNEUROSCI.16-06-02027.1996, PMID: 8604047 PMC6578509

[ref29] Lau A. A. Crawley A. C. Hopwood J. J. Hemsley K. M. (2008). Open field locomotor activity and anxiety-related behaviors in mucopolysaccharidosis type IIIA mice. Behav. Brain Res. 191, 130–136. doi: 10.1016/j.bbr.2008.03.024, PMID: 18453006

[ref30] Lazarov O. Mattson M. P. Peterson D. A. Pimplikar S. W. van Praag H. (2010). When neurogenesis encounters aging and disease. Trends Neurosci. 33, 569–579. doi: 10.1016/j.tins.2010.09.003, PMID: 20961627 PMC2981641

[ref31] Magnusson K. R. Scruggs B. Aniya J. Wright K. C. Ontl T. Xing Y.. (2003). Age-related deficits in mice performing working memory tasks in a water maze. Behav. Neurosci. 117, 485–495. doi: 10.1037/0735-7044.117.3.485, PMID: 12802877

[ref32] Malatynska E. Steinbusch H. W. Redkozubova O. Bolkunov A. Kubatiev A. Yeritsyan N. B.. (2012). Anhedonic-like traits and lack of affective deficits in 18-month-old C57BL/6 mice: implications for modeling elderly depression. Exp. Gerontol. 47, 552–564. doi: 10.1016/j.exger.2012.04.010, PMID: 22583982

[ref33] Marques F. Mesquita S. D. Sousa J. C. Coppola G. Gao F. Geschwind D. H.. (2012). Lipocalin 2 is present in the EAE brain and is modulated by natalizumab. Front. Cell. Neurosci. 6:33. doi: 10.3389/fncel.2012.00033, PMID: 22907989 PMC3414908

[ref34] Martínez-Mota L. Ulloa R. E. Herrera-Pérez J. Chavira R. Fernández-Guasti A. (2011). Sex and age differences in the impact of the forced swimming test on the levels of steroid hormones. Physiol. Behav. 104, 900–905. doi: 10.1016/j.physbeh.2011.05.027, PMID: 21658399

[ref35] Mattson M. P. Magnus T. (2006). Ageing and neuronal vulnerability. Nat. Rev. Neurosci. 7, 278–294. doi: 10.1038/nrn1886, PMID: 16552414 PMC3710114

[ref36] Merkley C. M. Jian C. Mosa A. Tan Y. F. Wojtowicz J. M. (2014). Homeostatic regulation of adult hippocampal neurogenesis in aging rats: long-term effects of early exercise. Front. Neurosci. 8:174. doi: 10.3389/fnins.2014.00174, PMID: 25071426 PMC4077125

[ref37] Moyer J. R. Brown T. H. (2006). Impaired trace and contextual fear conditioning in aged rats. Behav. Neurosci. 120, 612–624. doi: 10.1037/0735-7044.120.3.612, PMID: 16768613

[ref38] Mu Y. Gage F. H. (2011). Adult hippocampal neurogenesis and its role in Alzheimer's disease. Mol. Neurodegener. 6:85. doi: 10.1186/1750-1326-6-85, PMID: 22192775 PMC3261815

[ref39] Mucha M. Skrzypiec A. E. Schiavon E. Attwood B. K. Kucerova E. Pawlak R. (2011). Lipocalin-2 controls neuronal excitability and anxiety by regulating dendritic spine formation and maturation. Proc. Natl. Acad. Sci. U. S. A. 108, 18436–18441. doi: 10.1073/pnas.1107936108, PMID: 21969573 PMC3215032

[ref40] Naude P. J. Eisel U. L. Comijs H. C. Groenewold N. A. De Deyn P. P. Bosker F. J.. (2013). Neutrophil gelatinase-associated lipocalin: a novel inflammatory marker associated with late-life depression. J. Psychosom. Res. 75, 444–450. doi: 10.1016/j.jpsychores.2013.08.023, PMID: 24182633

[ref41] Naude P. J. Nyakas C. Eiden L. E. Ait-Ali D. van der Heide R. Engelborghs S.. (2012). Lipocalin 2: novel component of proinflammatory signaling in Alzheimer's disease. FASEB J. 26, 2811–2823. doi: 10.1096/fj.11-202457, PMID: 22441986 PMC3382095

[ref42] Nicolle M. M. Gonzalez J. Sugaya K. Baskerville K. A. Bryan D. Lund K.. (2001). Signatures of hippocampal oxidative stress in aged spatial learning-impaired rodents. Neuroscience 107, 415–431. doi: 10.1016/s0306-4522(01)00374-8, PMID: 11718997

[ref43] Ottoni E. B. (2000). EthoLog 2.2: a tool for the transcription and timing of behavior observation sessions. Behav. Res. Methods Instrum. Comput. 32, 446–449. doi: 10.3758/bf03200814, PMID: 11029818

[ref44] Perna G. Iannone G. Alciati A. Caldirola D. (2016). Are anxiety disorders associated with accelerated aging? A focus on neuroprogression. Neural Plast. 2016:8457612. doi: 10.1155/2016/8457612, PMID: 26881136 PMC4736204

[ref45] Rapp P. R. Heindel W. C. (1994). Memory systems in normal and pathological aging. Curr. Opin. Neurol. 7, 294–298. doi: 10.1097/00019052-199408000-00003 7952236

[ref46] Shoji H. Takao K. Hattori S. Miyakawa T. (2016). Age-related changes in behavior in C57BL/6J mice from young adulthood to middle age. Mol. Brain 9:11. doi: 10.1186/s13041-016-0191-9, PMID: 26822304 PMC4730600

[ref47] Small S. A. Tsai W. Y. DeLaPaz R. Mayeux R. Stern Y. (2002). Imaging hippocampal function across the human life span: is memory decline normal or not? Ann. Neurol. 51, 290–295. doi: 10.1002/ana.10105, PMID: 11891823

[ref48] van Praag H. Shubert T. Zhao C. Gage F. H. (2005). Exercise enhances learning and hippocampal neurogenesis in aged mice. J. Neurosci. 25, 8680–8685. doi: 10.1523/JNEUROSCI.1731-05.2005, PMID: 16177036 PMC1360197

[ref49] Wu M. V. Luna V. M. Hen R. (2015). Running rescues a fear-based contextual discrimination deficit in aged mice. Front. Syst. Neurosci. 9:114. doi: 10.3389/fnsys.2015.00114, PMID: 26321926 PMC4531235

[ref50] Yin F. Sancheti H. Patil I. Cadenas E. (2016). Energy metabolism and inflammation in brain aging and Alzheimer's disease. Free Radic. Biol. Med. 100, 108–122. doi: 10.1016/j.freeradbiomed.2016.04.200, PMID: 27154981 PMC5094909

